# Phage/nanoparticle cocktails for a biocompatible and environmentally friendly antibacterial therapy

**DOI:** 10.1007/s00253-025-13526-x

**Published:** 2025-05-29

**Authors:** Mateusz Wdowiak, Sada Raza, Mateusz Grotek, Rafał Zbonikowski, Julita Nowakowska, Maria Doligalska, Ningjing Cai, Zhi Luo, Jan Paczesny

**Affiliations:** 1 Institute of Physical Chemistry, Polish Academy of Sciences, Marcina Kasprzaka 44/52, 01-224 Warsaw, Poland; 2 Centre of New Technologies, University of Warsaw, Stefana Banacha 2 C, 02-097 Warsaw, Poland; 3 Military University of Technology, Gen. Sylwestra Kaliskiego 2, 00-908, Warsaw, Poland; 4 Faculty of Biology, University of Warsaw, Ilii Miecznikowa 1, 02-096 Warsaw, Poland; 5 Laboratory of Bioinspired Medicine and Materials, Southern University of Science and Technology, 1088 Xueyuan Avenue, Shenzhen, 518055 People’s Republic of China

**Keywords:** Silver nanoparticles, Bacteriophages, Antimicrobial agents, Antimicrobial combinations, *Acanthamoeba castellanii*

## Abstract

**Abstract:**

Antibiotic resistance continues to rise, necessitating alternative strategies. Bacteriophages have emerged as promising natural antibacterial agents, offering a targeted approach to combating bacterial infections. Combining bacteriophages with nanoparticles presents a novel approach that could enhance antibacterial potency while reducing the risk of resistance. While phage/antibiotic cocktails are widely explored to enhance antibacterial efficacy and prevent resistance, research on phage/nanoparticle combinations remains limited. We explore the synergy between green tea extract-capped silver nanoparticles (G-TeaNPs) and bacteriophages in combating pathogenic bacteria (methicillin-resistant *Staphylococcus aureus*, *Salmonella enterica*). G-TeaNPs show minimal antiphage activity, ensuring compatibility in phage-NP formulations. These combinations significantly reduce bacterial counts in a short time (only 3 h), e.g., *S. aureus* survival is around 30% after incubation with just 0.001 mg/mL of G-TeaNPs, while G-TeaNPs and phages alone result in around 80% and 70% survival, respectively. Cytotoxicity tests against eukaryotic 3T3 NIH fibroblast cells confirm biocompatibility at effective concentrations. Additionally, we examine G-TeaNPs’ impact on the free-living protist *Acanthamoeba castellanii*. Both green tea extract and G-TeaNPs can reduce *A. castellanii* cell counts by 80%, but only at high concentrations. Microscopy revealed nanoparticle uptake by amoebae, causing intracellular accumulation and vacuolization, while green tea extract induced similar changes without uptake. Our findings highlight G-TeaNPs as safe, effective agents in phage/nanoparticle antibacterial formulations with dual antimicrobial and amoebicidal properties for therapeutic and environmental applications.

**Keypoints:**

• *Silver nanoparticles synthesized with tea extracts (G-TeaNPs) have a minimal effect on the tested viruses.*

• *Combining G-TeaNP with bacteriophages offers new-generation antibacterial cocktails.*

• *Green tea extracts and AgNPs present concentration-dependent anti-amoebic activity.*

**Supplementary Information:**

The online version contains supplementary material available at 10.1007/s00253-025-13526-x.

## Introduction

Phages eliminate approximately 40% of bacterial biomass daily (Ranveer et al. 2024). However, research on the effects of phages on cohabiting microorganisms remains limited and is often overlooked, even in complex ecosystems (Czajkowski et al. 2019). While phages have been a focal point of studies in environments such as food processing, the human gut, and plant crops, much remains unknown about the role of environmental phages and their impact across different contexts (Ranveer et al. 2024). Although the use of lytic phages to control bacterial pathogens (phage therapy) for nearly a century, the recent rise of antibiotic-resistant bacteria has sparked renewed interest in phage research. Phage therapy, particularly in clinical and veterinary settings, has gained attention for its therapeutic and antibacterial potential (Rai et al. 2024). More broadly, phages have been used as biological control agents to reduce bacterial loads in various contexts, such as targeting *Listeria monocytogenes* in food processing, controlling zoonotic pathogens in food animals, and treating crops for plant pathogenic bacteria (Abedon et al. 2017). Since the twenty-first century, research has significantly advanced our understanding of phage biology and immunology, ensuring the safety of phage therapy through modern technologies like whole-genome sequencing and automation (Kortright et al. 2019). Phage therapy is now used to treat various infections, with FDA-approved clinical trials showing no safety concerns. Renewed research and ongoing trials have demonstrated the efficacy and safety of phage therapy, offering a promising solution to the growing threat of antibiotic-resistant infections (Massoud et al. 2015; Maimaiti et al. 2023). The main disadvantage concerns identifying appropriate lytic phages with high virulence and a broad-spectrum of bacterial hosts to suit different patients (Zalewska-Piątek 2023). Several other factors contribute to the infrequent commercialization of phage preparations, including high costs, challenges in patenting (Lin et al. 2021), and the absence of regulatory approval for large-scale use (Jault et al. 2018).

While, despite its promising potential, phage therapy has not received widespread regulatory approval for human use, nanotechnology offers a more favorable approach to overcoming several challenges associated with phage preparations. Advances in nanotechnology, such as nano-encapsulation, provide solutions to pharmacological and clinical obstacles by shielding phages from the host immune system and environmental factors, ensuring controlled release, and enhancing delivery efficiency (Kaur et al. 2021). Encapsulation can form nanovesicles that shield phages and provide a controlled release (González-Menéndez et al. 2018). Nanofibers produced through electro-spinning offer another method to enhance phage therapy outcomes (Kaur et al. 2021). Integrating nanotechnology with phage therapy optimizes the pharmacokinetic profile of phages, ensuring better therapeutic efficacy and overcoming the limitations of phage-only formulations.

Phages are often tested as cocktails with antibiotics (Kim et al. 2024). By combining the precision of bacteriophages, which selectively target and lyse specific bacterial strains, with the broad-spectrum efficacy of antibiotics, these cocktails enhance bacterial eradication while reducing the likelihood of resistance development. Phages can disrupt bacterial biofilms, increasing antibiotic penetration and effectiveness (Kebriaei et al. 2023), while antibiotics can weaken bacterial defenses, making them more susceptible to phage attacks (Akturk et al. 2023). There are also attempts to mix phages with other adjuvants, e.g., essential oils (Elafify et al. 2025).

Not many reports show the simultaneous action of phages and nanoparticles. For example, a combination of silver nanoparticles (AgNPs) and bacteriophage ZCSE2 demonstrated enhanced antibacterial properties against *Salmonella*, with effective MIC and MBC values at 23 μg/mL (Abdelsattar et al. 2021). Similarly, gold nanorods conjugated with chimeric M13 phages, forming “*phanorods*,” effectively targeted *P. aeruginosa* biofilms in wound infections, highlighting their potential for treating superficial infections (Pardo-Freire and Domingo-Calap 2023). Another study synthesized Ag-CS nanoparticles with phages, achieving significant bactericidal and inhibitory effects against gram-positive and gram-negative bacteria and biofilm production with MIC values between 31.2 and 62.2 μg/mL and MBC values between 15.6 and 500 μg/mL (Abdelsattar et al. 2023). Additionally, T7 phages armed with silver nanoparticles efficiently eradicated bacterial biofilms and were non-toxic to eukaryotic cells, indicating a synergistic and safe strategy for biofilm treatment (Szymczak et al. 2024; Szymczak and Golec 2024). Beyond bacterial infections, the integration of therapeutic viruses and nanomaterials has also revolutionized cancer therapy, enabled precision targeting, and reduced side effects, thus presenting a novel approach to overcoming the limitations of traditional treatments (Li et al. 2023). These examples underscore the potential of phage/nanoparticle combinations as potent antimicrobial and therapeutic agents.

As described in our previous study tea extracts, with emphasis on green tea, are characterized by a rich composition of flavanols and polyphenols, including catechins as one of the major classes of polyphenolic compounds. Polyphenols serve as both reducing and capping agents during nanoparticle synthesis. In the presence of metal ions, the hydroxyl groups of catechin molecules act as electron donors, reducing silver ions (Ag^+^) to metallic silver (Ag^0^). Subsequent interactions between polyphenols and the resulting silver nanoparticles (AgNPs) facilitate surface capping, which prohibits agglomeration and contributes to the overall stability of the preparation (Rolim et al. 2019; Raza et al. 2023). Additionally, tea extracts-capped nanoparticles are recognized for their superior antimicrobial activity compared to “traditional” silver nanoparticles (Xin et al. 2022; Fahim et al. 2024; Shahzadi et al. 2025).

In this study, we verified whether metal NPs could be effectively used against viruses. We confirmed that AgNPs, including NPs synthesized using green tea extracts, had no significant “antiphagent” activity towards the examined viruses. This observation allowed us to prepare phage/NP cocktails of superior activity toward bacterial pathogens. These formulations presented improved antibacterial activity compared to frequently used phage/antibiotic cocktails. Finally, using mammalian cells and the free-living unicellular organism *A. castellanii*, we proved that our nanoparticles were safe at dedicated concentrations for both humans and aquatic protists. Additionally, we explained the potential amoebicidal activity of green tea extracts and G-TeaNPs.

## Materials and methods

### Synthesis of C-AgNPs and G-TeaNPs

All commercially available reagents were used as received without further purification. According to the product specifications provided by the manufacturer (Sigma-Aldrich), reagent purity exceeded 98%, as determined by gas chromatography (GC) analysis or titration with appropriate reagents. A control batch of silver nanoparticles was synthesized following a modified version of the method by Agnihotri et al. (Agnihotri et al. 2014). In short, an aqueous solution (48 mL) containing 2 mM NaBH_4_ and 2 mM trisodium citrate (TSC) was heated to 60 °C and stirred for 30 min. Next, the AgNO_3_ solution (2 mL, 11.7 mM) was added dropwise. The mixture was heated to 90 °C, and the pH was adjusted to 10.5 using 0.1 M NaOH. Finally, the reaction was stirred further at 90 °C for 20 min until an evident color change was observed.

Green tea silver nanoparticles (G-TeaNPs) were synthesized using a slightly modified method by Nakhjavani et al. (Nakhjavani et al. 2017), according to the protocol described in our previous study (Raza et al. 2023). In short, green tea leaves (Loyd, Mokate, Poland) were frozen with liquid nitrogen and ground into a fine powder using a mortar and pestle. To prepare the extract, 10 g of the ground tea leaves were brewed in 100 mL of hot distilled water (60 °C) for 15 min. The extract was then cooled to room temperature, centrifuged twice (9000 rpm for 10 min, followed by 15,000 rpm for 10 min) to remove debris, and filtered using a 0.22 μm syringe filter. For nanoparticle synthesis, 12 mL of the filtered extract was mixed with 13 mL of ultrapure water and stirred in an Erlenmeyer flask while 750 μL of 10 mM AgNO_3_ was added dropwise. The reaction mixture was stirred for 2 h, during which the solution’s color turned yellow–brown with a silver shine, indicating nanoparticle formation. The resulting suspension was centrifuged at 10,000 rpm for 10 min, and the pellet was redispersed in distilled water. This purification step was repeated six times. Finally, the nanoparticles were suspended TM buffer solution at a concentration of 1 mg/mL and stored at 4 °C for later use.

### DLS and zeta potential

Dynamic light scattering (DLS) and zeta potential measurements were determined using a Zetasizer Nano ZS (Malvern Instruments Ltd., Malvern, UK) equipped with a DLS module (He–Ne laser 633 nm, max 4 mW, allowing for measuring backscattered (175°) and forward-scattered (12.8°) light). Quasi-backscattered light was used for the measurements. One centimeter quartz cuvettes (Hellma, Germany) were used.

### Scanning electron microscopy (SEM) and energy-dispersive X-ray spectroscopy (EDX)

Scanning electron microscope (SEM) imaging was performed using a Nova NanoSEM 450 under high vacuum conditions (~ 10^−7^ mbar). Samples were deposited onto a silicon substrate, air-dried, and affixed to a standard SEM stub using carbon tape. Imaging was conducted with the Through-Lens Detector (TLD) in secondary electron mode at a primary beam energy of 10 kV and a working distance of 4.8 mm. Nanoparticle (NP) diameters were quantified from SEM images using ImageJ software, with measurements obtained from a minimum of 100 particles per sample. Energy-dispersive X-ray spectroscopy (EDX) was executed simultaneously with SEM observations for chosen regions of the samples.

### Fourier-transformed infrared spectroscopy (FTIR)

Fourier-transform infrared (FTIR) spectroscopy was performed using a Vertex 80 V FTIR spectrometer (Bruker, USA) equipped with a Platinum ATR module (Bruker, USA). Tea extracts were dried by rotary evaporation at 65 °C to remove water, while G-TeaNPs were centrifuged to eliminate residual water and subsequently dried in an oven at 65 °C. The dried powder samples were placed onto a diamond prism (1 mm × 1 mm) to ensure complete coverage. Spectral measurements were conducted at a resolution of 2 cm^−1^, with a total of 64 scans collected per sample.

### PXRD

X-ray diffraction analysis was performed with a Malvern PANalytical Empyrean range diffractometer at room temperature, with a wavelength *λ* = 0.154 nm.

### Bacteriophage-AgNP cocktails against bacterial pathogens

The bacteria were cultured according to the plating method protocol. The overnight cultures were refreshed by adding 7.5 mL of fresh LB medium to 2.5 mL of overnight culture and incubated for one hour at 37 °C. Then, the refreshed cultures were both diluted with LB to reach an optical density of around OD_600_ = 1.0 for *Salmonella enterica* DSM 18552 (about 8.0 × 10^8^ CFU/mL) (colony-forming units per mL), OD_600_ = 1.0 for *Staphylococcus aureus* ATCC 43300 (about 5.0 × 10^9^ CFU/mL). Afterward, the bacteria were centrifuged (10 min, 8200 RPM) and suspended in a PBS buffer solution to reach the initial concentration of 10^5^ CFU/mL. Then, bacteria were exposed to varying rates of infection (ROI) of adequate phages (P22, vB_SauS_CS1) of 1, 10, and 100, and concentrations of G-TeaNPs ranging from 0.1 to 0.0001 mg/mL. Both P22 and vB_SauS_CS1 were purchased from the German Collection of Microorganisms and Cell Cultures.

The survival rate was evaluated after 3 h of incubation with mixing (220 RPM, 37 °C). After the incubation, 100 mL of each sample was placed onto the fresh LB agar plates. The plates were incubated overnight at 37 °C. Then, the number of bacteria was calculated based on the colony number, according to the equation CFU per mL = N × D × 10 (N – number of colonies; D – dilution).

### Toxicity assay on Acanthamoeba castellanii

The cytotoxic effect of reagents such as AgNPs, G-TeaNPs, and green tea extract was tested in vitro on strain 309 of *A. castellanii* T4 genotype (isolated from the environment (Kasprzak and Mazur 1978)). Amoeba strains were cultured axenically on Bacto-Casitone liquid medium + horse serum as described by Červ (L. 1969). Amoeba cultures were established on polystyrene plates (Nest Biotechnology Co., Ltd., Non-Pyrogenic) with 24 wells. One milliliter of cell suspension was added to the wells, and 1 mL of reagent containing a concentration of AgNPs, green tea extract, and G-TeaNPs to achieve the target dilution. Reagents were added to the axenic amoeba culture (5 × 10^4^ cells/mL) in the following concentrations: 0.05, 0.1, 5, 10, and 20 mg/mL. The increase or decrease in the number of amoebae was checked at 48-h intervals using Thom’s hemocytometer chamber. The control group was a culture of amoebae without reagents (Derda et al. 2016). The minimum concentration of reagents that inhibited the growth of the amoeba culture was determined. The test was performed in 3 replicates for the same batch of particles, up to 7 days, of cultures stored at 28 °C in a humid and dark chamber.

### Transmission electron microscope (TEM) imaging

The cells were fixed in 2.5% glutaraldehyde in 0.1 M cacodylate buffer, pH 7.2, overnight at room temperature. Samples were washed in cacodylate buffer thrice and stained with 1% osmium tetroxide in ddH_2_O overnight at room temperature. Samples were then washed in ddH_2_O and dehydrated through a graded series of ethanol (30%, 50%, 70%, 80%, 96%, absolute ethanol, and acetone). Samples were embedded in epon resin (SERVA) and polymerized for 24 h at 60 °C in an incubator (Agar Scientific, England). Next, 70 nm sections were cut with a diamond knife on RMC MTXL ultramicrotome (RMC Boeckeler Instruments, USA). The sections on copper grids were not contrasted. Samples were analyzed using a LIBRA 120 transmission electron microscope produced by Carl Zeiss (Oberkochen, Germany) at 120 keV. Photographs were made with a Slow-Scan CCD camera (ProScan, Germany) using the EsiVision Pro 3.2 software.

### Cytotoxicity towards mammalian cells

The biocompatibility of modified gold nanoparticles was assessed using the Alamar Blue assay using 3 T3 NIH fibroblast cells. All reagents were obtained from commercial suppliers: AlamarBlue reagent (ThermoFisher Scientific), Triton X-100 (Sigma-Aldrich), and Trypan Blue (ThermoFisher Scientific). 3 T3 NIH fibroblast cells were cultured in Dulbecco’s Modified Eagle Medium (DMEM) supplemented with 10% fetal bovine serum (FBS) and 1% penicillin–streptomycin, in a humidified atmosphere at 37 °C and 5% CO_2_. Cells were subcultured upon reaching 70–80% confluency and harvested for experiments during the log phase of growth. Cells were harvested and counted using Trypan Blue exclusion to ensure viable cell counts. Cells were diluted to a concentration of 7.5 × 10 cells/mL, and 200 μL of cell suspension (equivalent to 1.5 × 10^4^ cells) was seeded into each well of a 96-well tissue culture-treated plate. The cells were incubated overnight at 37 °C with 5% CO_2_ to allow for surface attachment. Cells were treated with nanoparticles at a 0.1 mg/mL concentration the following day. The cells were incubated with the nanoparticles for 24 and 48 h at 37 °C in a humidified atmosphere with 5% CO_2_. After each incubation period, the medium was aspirated and replaced with fresh medium containing 10% (v/v) Alamar Blue solution. The cells were incubated with the AlamarBlue mixture for 4 h at 37 °C with 5% CO_2_. After incubation, 150 μL of the medium from each well was transferred to a new 96-well plate, and fluorescence was measured using a plate reader with excitation at 530—570 nm and emission at 580–620 nm. The blank value (from wells without cells) was subtracted from each reading to ensure accuracy. Cell viability was calculated based on metabolic activity, which was directly proportional to the Alamar Blue fluorescence intensity.

Cytotoxicity tests were also performed using MTT proliferation/metabolic activity assays, as described in the *Supplementary Information.*

### Analysis of the data

All the experiments were performed in triplicate (*n* = 3). The error bars were plotted as the standard error of measurement (SEM). The statistical analysis was performed using a *t*-test, with statistical significance marked as follows: ***p < 0.001, **p < 0.01, *p < 0.05. Otherwise, the differences were not significant (ns). In the Alamar blue cytotoxicity assay, where two independent variables (nanoparticle type and concentration) were involved, we employed a two-way ANOVA analysis; statistical significance was marked similarly as in the case of the *t*-test.

## Results

### Characterization of G-TeaNPs

**Fig. 1:**
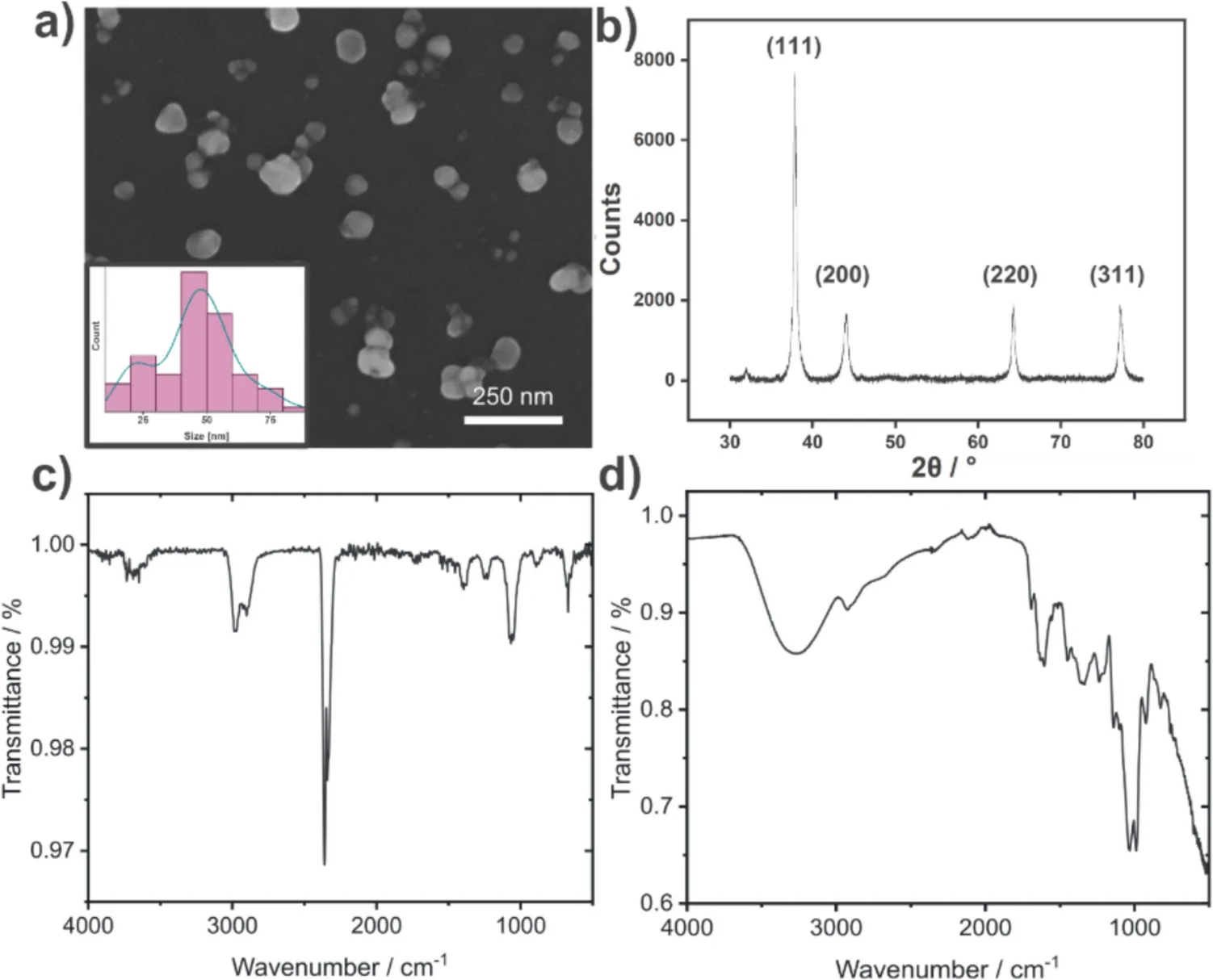
**a** SEM image of G-TeaNPs (silver nanoparticles synthesized using green tea extract), inset: size distribution (diameter), line—kernel density. **b** Diffractogram of AgNPs capped with green tea extract. **c** FTIR spectra of G-TeaNPs and **d** green tea extract

The SEM image of the silver nanoparticles (AgNPs) capped with green tea extract (G-TeaNPs) revealed their morphology, with agglomerates bound by a thin layer likely originating from the tea extract coating (Fig. 1a). The size distribution of the nanoparticles (Fig. 1a, insert) showed that most particles fell within the 40–50-nm range. However, a noticeable deviation from a normal distribution was observed due to an overrepresentation of particles around ~ 20 nm. The average particle diameter was calculated to be 45.6 ± 15.6 nm. This irregular size distribution was consistent with findings from our previous study, where G-TeaNPs’ efficacy against bacteria was evaluated (Raza et al. 2023). DLS measurements revealed the hydrodynamic diameter to be around 60–70 nm, which is in line with SEM. Zeta potential was negative. Detailed distributions of hydrodynamic diameters and zeta potentials are provided in the *Supplementary Information* (Figure S4).

The nanoparticles were further analyzed using Fourier-transform infrared spectroscopy (FTIR), revealing spectral features consistent with those reported in our previous study (Fig. 1c and d). A broad band in the 3000–3600 cm^−1^ range, characteristic of O–H stretching, was observed. This band is typical of alcohols, phenols, and carboxylic acids commonly found in tea extract components, and possibly due to adsorbed water molecules. Additional prominent bands included C–H stretching (2850–2950 cm^−1^), C = O stretching (1700–1750 cm^−1^), C = C stretching (1620–1680 cm^−1^), N–H bending (1500–1600 cm^−1^), and C–O stretching (1050–1300 cm^−1^). These bands corresponded to functional groups commonly found in tea extract constituents, such as catechins, flavonoids, phenolic acids, and amino acids. Notably, except for the broad O–H stretching band, the same characteristic peaks were observed in the FTIR spectrum of the G-TeaNPs, confirming the successful capping of nanoparticles by the tea extract components.

Energy-dispersive X-ray spectroscopy (EDX) was performed on the AgNPs sample, with the Si substrate used as a reference background. The analysis confirmed the presence of silver in the sample (Figure S1). The X-ray diffraction (XRD) analysis of the sample displayed a characteristic diffractogram of AgNPs, i.e., the face-centered cubic crystalline structure of metallic silver (JCPDS 01–071–4613) (Fig. 1b). Using Scherrer’s equation, the crystallite size was calculated from the four prominent diffraction peaks (with a shape factor of 0.94), yielding an average size of 19.8 ± 3.4 nm. This suggests that a significant portion of the nanoparticles were not single crystals, which might also account for the overrepresentation of ~ 20 nm particles observed in the size distribution analysis. We also analyzed two peaks at 32.0° and 35.9° of low intensity. They were associated with the highly intense peaks of silver oxide species, such as Ag_2_O, AgO, and Ag_2_O_3_ (Ando et al. 2012). These findings indicated that the oxidation of the silver nanoparticles during synthesis or storage was minimal.

### Antimicrobial activity of G-TeaNPs

Before testing phage/nanoparticle cocktails against pathogenic bacteria, evaluating the potential effects of the nanoparticles (NPs) on phages is essential. Figure S2 confirmed that our nanoparticles exhibited no significant inactivation effects on the tested phages. Taking into account “the experimental effect,” we subjectively acknowledged the decrease in phage titer > 1 log as the antiviral activity. While LR1_PAO1 showed a minor reduction of approximately 0.5 log compared to the control group containing only phages, there was no observable difference between the effects of citrate-capped nanoparticles and tea-based nanoparticles on LR1_PAO1. This suggested that any slight reduction in PFU/mL was not attributable to the functionalization of the tea extract. For T4 and Phi6, no measurable decrease in phage activity was observed for any of the tested nanoparticles, indicating the lack of antiviral activity of AgNPs, including the G-TeaNPs, against both enveloped and non-enveloped viruses. These results indicated that the nanoparticles, whether citrate-capped or tea-capped, did not possess phage-inactivating properties, supporting their compatibility for further testing in phage/nanoparticle combinations targeting pathogenic bacteria. We proceeded to test phage/nanoparticle cocktails using green tea silver nanoparticles. G-TeaNPs were previously identified as the most effective against bacteria and fungi among a series of silver particles prepared with extracts from various teas (Raza et al. 2023).

**Fig. 2:**
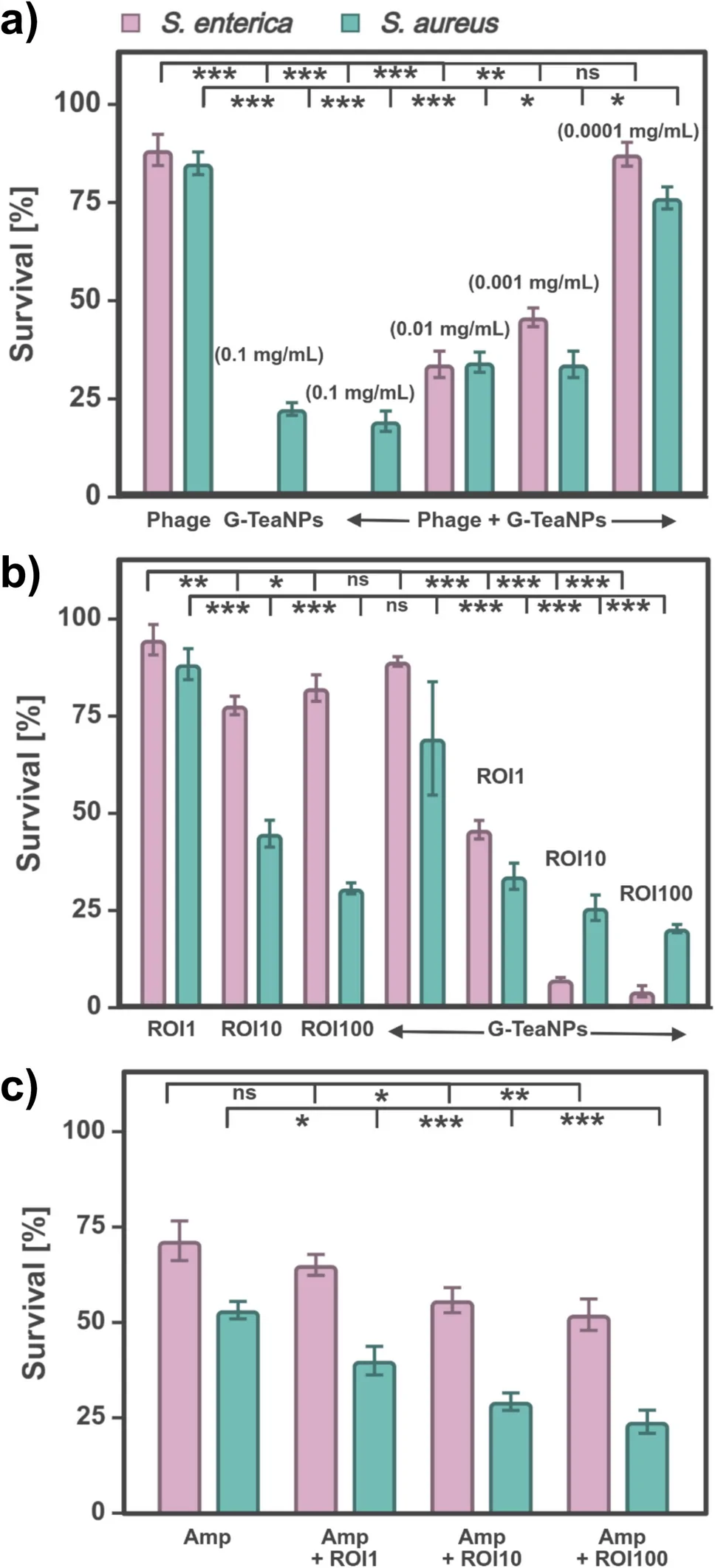
Antibacterial activity of phage/nanoparticle (phage/NP) cocktails and phage/antibiotic combinations against *S. aureus* (gram-positive) and *S. enterica* (gram-negative). **a** Bacterial survival after treatment with G-TeaNPs (0.1 mg/mL to 0.0001 mg/mL) combined with P22 (*S. enterica*) or vB_SauS_CS1 (*S. aureus*) bacteriophages (ROI = 1). **b** Effect of phage/NP cocktails ([G-TeaNPs] = 0.001 mg/mL) at different rates of infection (ROI = 1 to 100). **c** Comparison of bacterial survival following treatment with ampicillin (AMP) alone or in combination with bacteriophages at varying ROIs

For the novel phage/NP cocktails, we selected *S. enterica*, one of the most frequently occurring foodborne pathogens (a gram-negative bacterium), and a methicillin-resistant strain of *S. aureus* (MRSA; a gram-positive bacterium). The efficacy of these combinations was assessed across a range of rates of infection (ROI, representing the ratio of phages to bacterial cells) and concentrations. In Fig. 2a, ROI = 1 was kept constant, but the concentration of G-TeaNPs varied from 0.1 mg/mL to 0.0001 mg/mL. Figure 2 b shows the results of experiments where the concentration of G-TeaNPs was constant (0.001 mg/mL), but ROIs varied from 1 to 100.

Adding phages at an ROI = 10 or higher significantly enhanced antibacterial effects, allowing for a substantial reduction in the required working concentrations of G-TeaNPs. Specifically, with phages at ROI = 10, the bacterial survival rate decreased to 10–20%, depending on the species, even when the concentration of G-TeaNPs was as low as 0.001 mg/mL (1 μg/mL). In contrast, a similar efficacy with G-TeaNPs alone required a 100 times higher concentration (0.1 mg/mL).

For comparison with commonly used phage/antibiotic cocktails, we tested the combined effects of bacteriophages and ampicillin (AMP) against *S. aureus* and *S. enterica* across various rates of infection (ROIs). The results showed that the AMP-phage combination was more effective against *S. aureus* (Fig. 2d) compared to *S. enterica*. Specifically, AMP alone reduced *S. aureus* survival to approximately 60%, while adding phages further reduced survival to around 30% at ROI 10 and 25% at ROI 100. In the case of *S. enterica*, the impact of the AMP-phage combination was less pronounced but still significant. AMP alone decreased bacterial survival to approximately 75%. Adding phages reduced survival to around 70% (AMP + ROI 1) and 58% (AMP + ROI 100). Notably, the phage/NP cocktails at ROIs 10 and 100 achieved far greater reductions in survival, with bacterial survival rates dropping to approximately 10%.

The overall efficacy of the AMP-phage combination was similar to or worse than that of the phage/NP cocktails. This might be crucial when fighting multidrug-resistant bacteria, where antibiotics are inefficient and novel agents are needed. We also underline that these results were obtained after only 3 h of incubation.

### Cytotoxicity towards amoebae and mammalian cells

**Fig. 3:**
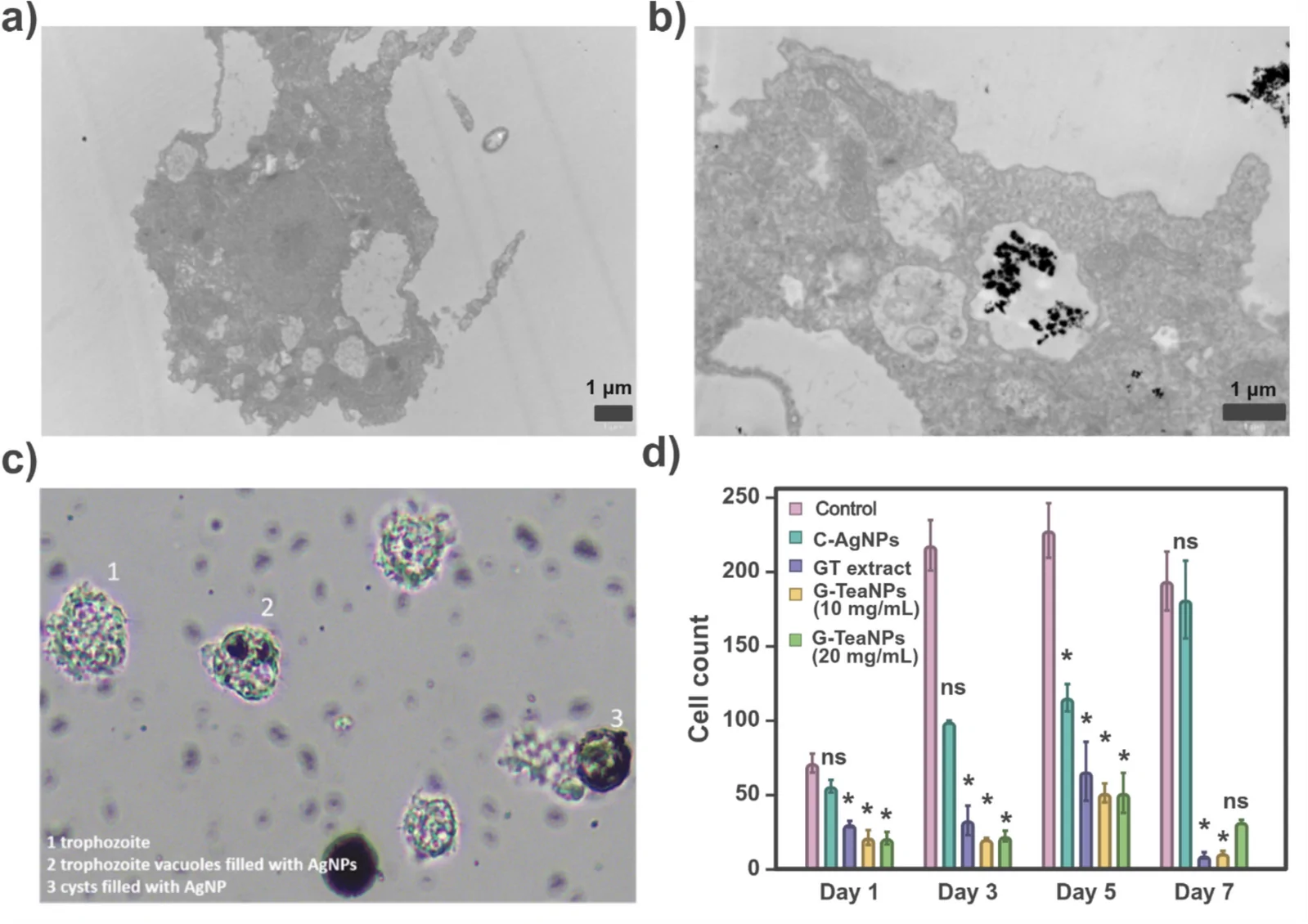
**a** Control cells of *A. castellanii*, non-exposed to G-TeaNPs. **b** *A. castellanii* exposed to G-TeaNPs, showing nanoparticle uptake and accumulation within intracellular vacuoles, compared to control cells. **c** Cells exposed to G-TeaNPs exhibit increased vacuolization and morphological changes: (1) trophozoites, (2) trophozoite vacuoles filled with AgNPs, and (3) cysts containing AgNPs. Similar vacuolization and size/shape reduction were observed in cells treated with green tea extract but without nanoparticle accumulation. These findings highlight the cellular uptake of nanoparticles and their potential influence on *A. castellanii* morphology. **d** Cytotoxic effects of citrate-capped C-AgNPs, G-TeaNPs, and green tea extract (GT) on *A. castellanii* over 7 days. G-TeaNPs inhibited amoebae growth only at high concentrations, while lower concentrations (i.e., 0.1 mg/mL as used in the other assays) were ineffective. GT alone was as effective as G-TeaNPs in reducing amoebae counts

To assess potential environmental impacts and effects on free-living unicellular organisms, *Acanthamoeba castellanii* was used as a model organism. Amoebae were exposed to tea extracts, citrate-capped AgNPs, and G-TeaNPs for seven days, with live amoebae counts taken every two days (Fig. 3d). The experiments revealed that G-TeaNPs were ineffective against *A. castellanii* at low concentrations. Depending on the batch of particles, up to 5 mg/mL did not affect amoebae. In the case of the specific batch shown here, the lowest concentration inhibiting the growth was 10 mg/mL, a dose approximately 16 times higher than the cytotoxic threshold for mammalian cells. Interestingly, no clear correlation was observed between G-TeaNP concentration and the formation of amoebae cysts. Surprisingly, green tea extracts alone were as effective in inhibiting amoebae growth as G-TeaNPs, a phenomenon not observed in antibacterial assays.

Transmission electron microscopy confirmed that *A. castellanii* actively phagocytosed the nanoparticles, accumulating within intracellular vacuoles (Fig. 3b). Light microscopy further revealed distinct morphological changes in both live amoebae and cysts after exposure to G-TeaNPs (Fig. 3c). Specifically, trophozoites and cysts were visibly filled with AgNPs within their vacuoles after five days of exposure. Exposure to green tea extract alone also induced noticeable reductions in cell size and shape and increased vacuolization, similar to what was observed in cells treated with G-TeaNPs. However, no nanoparticle accumulation was observed in the case of green tea extract.

The control sample showed normal *A. castellanii* forms, including moving trophozoites, resting trophozoites, and cysts, while cells exposed to G-TeaNPs display increased vacuolization, with AgNPs accumulating inside trophozoites and cysts. Similar vacuolization and morphological changes were observed in cells treated with green tea extract, although without the visible presence of nanoparticles.

These findings highlight the cellular uptake of nanoparticles by *A. castellanii* and their potential influence on amoebae morphology. Additionally, the inhibitory effect of green tea extract on *A. castellanii* raises questions about its mechanism of action, independent of nanoparticles, and its broader implications for environmental and biological systems. Importantly, these results also underscore the species-specific effects of G-TeaNPs, with significant differences in cytotoxicity between mammalian cells and amoebae.

To evaluate the biocompatibility of G-TeaNPs nanoparticles, an Alamar Blue assay was conducted using the 3 T3 NIH fibroblast cell line. Two types of nanoparticles were tested: citrate-capped silver nanoparticles (AgNPs) and G-TeaNPs. The chosen concentrations were 1 mg/mL and 0.1 mg/mL, based on their effective antibacterial activity.

**Fig. 4:**
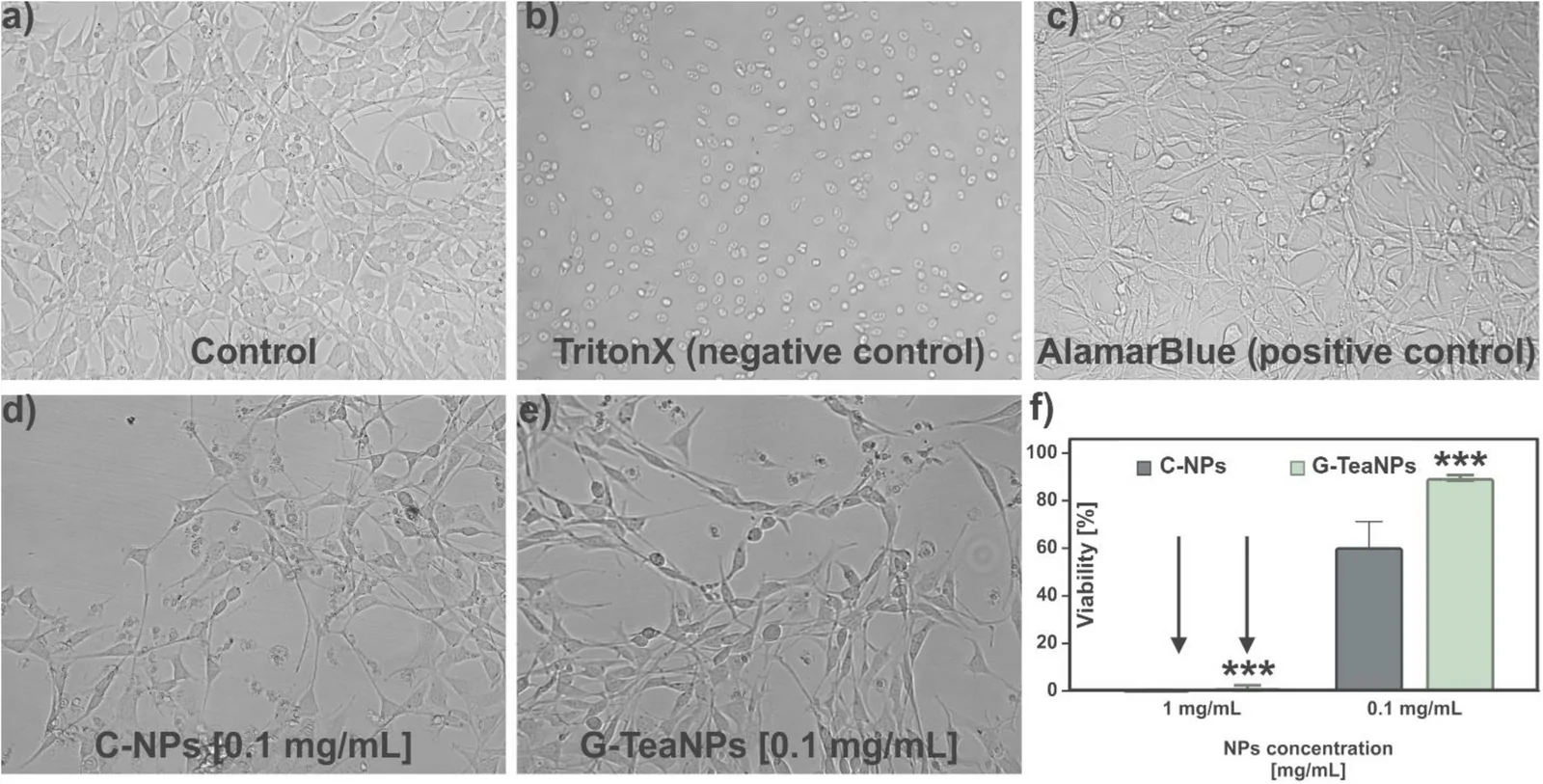
Optical microscopic images of 3 T3 NIH fibroblast cells after 24 h of incubation with different nanoparticle treatments: **a** untreated control cells. **b** Cells treated with 1% Triton X as a negative control. **c** Cells treated with only AlamarBlue (positive control). **d** Cells treated with control silver nanoparticles (C-AgNPs, 0.1 mg/mL), d) Cells treated with G-TeaNPs (0.1 mg/mL). **e** Viability of cells treated with AgNPs and G-TeaNPs after 24 h of incubation at 1 mg/mL and 0.1 mg/mL. **f** The survival of the fibroblasts after exposure to control AgNPs and G-TeaNPs at the concentrations of 1 mg/mL and 0.1 mg/mL. *p* < 0.001 by two-way ANOVA for both concentration and nanoparticle type, with significant interaction between factors

3 T3 NIH cells were treated with different nanoparticle formulations, including AgNPs and G-TeaNPs, for 24 h. Cell viability was assessed using the Alamar Blue assay, which measured the conversion of resazurin to its fluorescent product, resorufin. The quantitative results in Fig. 4f demonstrate that cell viability exceeded 80% for G-TeaNPs at 0.1 mg/mL, while that of AgNPs remained below 60%. At 1 mg/mL, both the nanoparticles showed cell viability below 5%.

Observations of cell morphology further confirmed nanoparticle biocompatibility. Untreated control cells (Fig. 4a) maintained their characteristic elongated, spindle-like morphology, typical of healthy 3 T3 fibroblasts. Similarly, cells exposed to AlamarBlue as a positive control (Fig. 4c) maintained their morphology. In contrast, cells treated with Triton X-100 (1%; Fig. 4b), i.e., negative control, showed spherical, shrunken morphologies indicative of cell death, confirming the assay’s sensitivity in detecting cytotoxic effects. Cells in the presence of AgNPs and GTNPs displayed no significant morphological changes compared to controls at 0.1 mg/mL (Fig. 4d–e). These results suggest that G-TeaNPs retain high biocompatibility with 3 T3 NIH cells, with cell viability consistently above 80%.

The cytotoxicity of citrate-capped AgNPs and G-TeaNPs was examined using the MTT metabolic activity assay on HeLa cells, with results summarized in Figure S3. G-TeaNPs demonstrated significantly higher toxicity compared to citrate-capped AgNPs across all tested concentrations. At 2.5 mg/mL, G-TeaNPs reduced HeLa cell survival to approximately 20%, while C-AgNPs allowed over 50% survival. Even at lower concentrations, such as 1.25 mg/mL and 0.625 mg/mL, G-TeaNPs showed consistently greater cytotoxic effects, with survival rates dropping below 70%, compared to C-AgNPs, where survival remained above 70%. Notably, the concentration of G-TeaNPs required to reach < 70% cell survival (0.625 mg/mL) was over six times higher than the working concentration used in antibacterial assays and more than 600 times higher than the amount of G-TeaNPs used in phage/nanoparticle cocktails. These findings highlight the higher cytotoxicity of G-TeaNPs to mammalian cells and the importance of optimizing doses to ensure antimicrobial efficacy while minimizing potential toxicity.

## Discussion

The quest for novel antibacterial agents, driven by the increasing drug resistance among bacteria, forces the modern-day scientific community to lean towards less popular and long-forgotten agents, such as nanoparticles and bacteriophages. Numerous studies have proved their efficacy separately, but also indicated the possibility of resistance mechanisms (Labrie et al. 2010; Matuła et al. 2019). Since bacteriophages seem to be unaffected by the metal nanoparticles, combining phages and “conventional” nanoparticles becomes an exciting approach, allowing for overcoming the high specificity of bacteriophages and decreasing the working concentration of silver. The efficacy of such combinations was confirmed in both gram-negative and gram-positive bacteria models (Abdelsattar et al. 2021, 2023; Elsayed et al. 2023, 2024). The fact that green-synthesized NPs present improved antimicrobial activity indicates they may be an even more effective addition to phage therapy.

This study demonstrated the potential of green tea extract-capped silver nanoparticles (G-TeaNPs) as multifunctional agents, combining antimicrobial efficacy with broad biological applicability. Detailed characterization confirmed their nanoscale morphology, irregular size distribution, and functional group composition, consistent with green tea extract capping. Despite minor deviations, such as partial oxidation and the presence of aluminum compounds from the tea extract, the nanoparticles were stable and well-suited for subsequent applications. G-TeaNPs did not exhibit direct phage-inactivating properties, ensuring their compatibility in phage/nanoparticle cocktails. These cocktails proved particularly effective against gram-negative bacteria, such as *S. enterica*, achieving bacterial survival rates of approximately 10% at low nanoparticle concentrations (0.001 mg/mL, i.e., 1 μg/mL). This highlights their ability to enhance antibacterial performance while minimizing nanoparticle usage. Compared to the recent studies on phage/AgNPs cocktails (Khalid Mohamed et al. 2021; Salih et al. 2024; Szymczak et al. 2024), we managed to decrease the working concentration of AgNPs to only 1 μg/mL. This improvement was possible because, for this assay, we used green tea-synthesized nanoparticles (G-TeaNPs), which were previously found to be more efficient in bacteria elimination than frequently used citrate-capped AgNPs (Nakhjavani et al. 2017; Raza et al. 2023).

Besides the enhanced antibacterial activity, compared to traditional antibacterial agents, phage/nanoparticle cocktails offer major advantages, including but not limited to:

i. reduced resistance development, hence phages specifically target bacteria and co-evolve with them, and silver nanoparticles exert broad-spectrum antibacterial effects through multiple mechanisms,
ii. improved formulation’s biocompatibility, since natural extracts and bacteriophages allow the reduction of the amount of silver used,
iii. environmental friendliness, since green synthesis avoids toxic reducing agents.

Cytotoxicity testing revealed the dose-dependent effects of G-TeaNPs on 3 T3 NIH, HeLa cells, and *A. castellanii*. Although G-TeaNPs were more toxic to mammalian cells compared to citrate-capped AgNPs, the effective doses used in antibacterial assays were substantially lower than the cytotoxic thresholds. Satisfyingly low cytotoxicity (cell survival rate > 70%) of the G-TeaNPs, at the concentrations used in our cocktails, was proved with both protocols we used, including the Alamar blue staining and the MTT assay. These observations strongly suggested that, due to the significant decrease in the working concentration of silver, combinations such as those proposed in this study have a tremendous potential in bacterial elimination in living systems.

Notably, this study was among the first to evaluate the activity of tea-synthesized AgNPs and tea extracts alone against the protists (here represented by *A. castellanii*). Previous studies most frequently evaluated the conjugates of AgNPs with amoebicidal compounds, such as tetrazole (Anwar et al. 2024) or terpene (Rajendran et al. 2024). Along with the evaluation of the anti-amoebic activity of G-TeaNPs, we discovered that green tea extracts alone displayed amoebicidal effects comparable to G-TeaNPs, raising questions about their intrinsic activity and environmental implications. This finding is adjusted to the trend in the current studies focused on determining the amoebicidal phytoproducts (Chaúque et al. 2025). Microscopy studies confirmed nanoparticle uptake and accumulation in amoebae, with associated morphological changes, while green tea extract induced similar changes without nanoparticle presence. This indicated that AgNPs might primarily carry tea extract components inside amoeba cells in such systems. Nevertheless, significantly higher concentrations of G-TeaNPs, compared to the experiments on bacteria, were needed to achieve the amoebicidal effect. This was in line with the current state of the art, hence the fungal and amoebic infections are generally more difficult to cure than bacterial infections (Nucci and Perfect 2008; Nakaminami et al. 2017), and require specialized drugs, most frequently the azoles (Qubais Saeed et al. 2024). Our study indicated that not only can G-TeaNPs be successfully used in antibacterial, antifungal, and anti-amoebic therapy (the following two require higher concentrations), but also that, in the case of accidental release to the environment, aquatic protists would not be significantly affected.

However, some potential limitations of phage cocktail therapy are still to be overcome. First of all, the high specificity of phages requires fast and reliable methods for the identification of bacteria. Otherwise, the antibacterial efficacy would be related to the action of the NPs only. The next challenge that can be identified, the scalability, is a frequent bottleneck for novel antibacterial therapy. In the case of phage/nanoparticle cocktails, both phage amplification and purification, and green synthesis of AgNPs would require optimization and application on an industrial scale. Additionally, updated regulations would be essential to successfully apply combinations of biological and inorganic antimicrobial agents, such as the cocktails proposed in this study. The matter of the delivery and patient compliance also requires further research and investigation. While topical applications (e.g., for wound infections) are more feasible, systemic use of phage/NP formulations would require careful pharmacokinetic studies to ensure safety and efficacy. Patient acceptance of virus- and nanoparticle-based therapies may also be limited due to unfamiliarity, necessitating effective communication and education strategies. We envision the first application in industry, e.g., for membrane sterilization, where the regulations are not as restrictive.

Overall, the results underscore the versatility of G-TeaNPs in bacterial and phage-based applications while emphasizing the importance of dose optimization to balance efficacy with cytotoxicity. These findings provide valuable insights into green tea-derived nanomaterials’ mechanisms and environmental impact, paving the way for future studies in targeted therapeutic and ecological applications.

## Supplementary Information

Below is the link to the electronic supplementary material.

Supplementary file1 (DOCX 1531 KB)

## Data Availability

The raw data required to reproduce these findings are available to download from https://doi.org/10.18150/YBVYR9.
