# Supplementary material for: Phage/nanoparticle cocktails for a biocompatible and environmentally friendly antibacterial therapy
Source: Appl Microbiol Biotechnol. 2025 May 29;109:129. doi: 10.1007/s00253-025-13526-x (PMC12122614; doi:10.1007/s00253-025-13526-x)
Supplement: Supplementary file 1 [file 253_2025_13526_MOESM1_ESM.docx]

**Phage-nanoparticle cocktails for a biocompatible and environmentally friendly antibacterial therapy**

Mateusz Wdowiak^1,2^, Sada Raza^1,*^, Mateusz Grotek^1,3^, Rafał Zbonikowski^1^, Hossein Maleki-Ghaleh^1^, Julita Nowakowska^4^, Maria Doligalska^4*^, Ningjing Cai^5^, Zhi Luo^5^, Jan Paczesny^1,*^

^1^ Institute of Physical Chemistry, Polish Academy of Sciences, Marcina Kasprzaka 44/52, 01-224 Warsaw, Poland

^2^ University of Warsaw, Centre of New Technologies, Stefana Banacha 2c, 02-097 Warsaw, Poland

^3^ Military University of Technology, gen. Sylwestra Kaliskiego 2, 00-908 Warsaw, Poland

^4^ University of Warsaw, Faculty of Biology, Ilii Miecznikowa 1, 02‐096 Warsaw, Poland

^5^ Laboratory of Bioinspired Medicine and Materials, Southern University of Science and Technology, 1088 Xueyuan Avenue, Shenzhen 518055, P.R. China

Corresponding authors: Jan Paczesny, jpaczesny@ichf.edu.pl; Sada Raza, sraza@ichf.edu.pl; Maria Doligalska, m.doligalska@uw.edu.pl

**Figure S1**. Energy-dispersive X-ray spectra of the AgNPs capped with citrate (control, **(a)**), its background measurement **(b)**, green tea extract **(c),** and its background measurement **(d)**.

*“Antiphagent” activity of the studied nanoparticles*

Before testing phage-nanoparticle cocktails against pathogenic bacteria, it is important to evaluate the potential effects of the nanoparticles (NPs) on phages. To this end, three different phages were selected: T4, representing one of the most abundant phages; LR1_PAO1, known for its relatively resistant nature; and Phi6, a model for highly resistant eukaryotic viruses. Bacteriophages were purchased from Phage Consultants, Poland, (T4), the German Collection of Microorganisms and Cell Cultures (Phi6), or isolated by us from the Baltic seawater (LR1_PAO1). The phages were exposed to citrate-capped silver nanoparticles (C-NPs) as a control, as well as silver nanoparticles synthesized using black tea (B-TeaNPs), green tea (G-TeaNPs), and red tea (R-TeaNPs), following the previous research.

The change in PFU/mL was recorded after 24 hours using a double-layer agar (DLA) assay. Petri plates were first filled with 20 mL of LB agar medium and left to solidify. 4 mL of top LB agar (prepared with liquid medium and 0.5% agar instead of 1.5% agar) was cooled to around 50 °C, mixed with 200 μL of the refreshed culture of the appropriate bacteria strain, and poured onto the plate. For the quantification of bacteriophages, a standard droplet assay was used. The number of plaques was counted after incubation of the plates at 37 °C for 24 h.

As shown in **Figure S2**, none of the nanoparticles exhibited significant inactivation effects on the tested phages. While LR1_PAO1 showed a minor reduction of approximately 0.5 log compared to the control group containing only phages, there was no observable difference between the effects of citrate-capped nanoparticles and tea-based nanoparticles on LR1_PAO1. This suggested that any slight reduction in PFU/mL was not attributable to the tea extract functionalization. For T4 and Phi6, no measurable reduction in phage activity was observed for any of the tested nanoparticles, indicating the lack of antiviral activity of AgNPs, including the TeaNPs, against both enveloped and non-enveloped viruses.

**Figure S2.** The effect of citrate-capped silver nanoparticles (C-NPs) and tea-extract-functionalized silver nanoparticles (B-TeaNPs, G-TeaNPs, and R-TeaNPs) on the viability of three phages: T4, LR1_PAO1, and Phi6. Phage titers are presented as plaque-forming units per milliliter (PFU/mL).

*Cytotoxicity of the studied nanoparticles*

In the MTT proliferation/metabolic activity assays, we used a cancer cell line - HeLa (cervical cancer). The MTT assay was performed using around 10,000 cells/well for both tested cell lines (controlled with Countess II Cell Counter). Cells were seeded into a 96-well plate (Greiner Bio-One) and incubated in an incubator for 24 hours at 37°C. Then, the medium was removed, and the tested formula (containing dye and polymer) at five different concentrations (double dilutions) was added to the fresh cell medium. The experiments were repeated five times for each concentration. After 6 hours of incubation, the medium was replaced with a culture medium including 1 mM 3-(4,5-dimethylthiazol-2-yl)-2,5-diphenyltetrazolium bromide (MTT reagent, Thermo Fischer Scientific). Cells were incubated with MTT reagent for 3 hours at 37°C. Then, solutions were replaced with DMSO and incubated for 10 minutes. The absorbance in each well was measured at 540 nm using a SpectraMax i3x MultiMode Microplate Reader with injectors (Molecular Devices). For each experiment, negative and positive controls were included. Negative controls contained 1% Triton-X 100, while positive controls were cells cultured under standard conditions without the tested formula.

**Figure S3.** Cytotoxicity of citrate-capped AgNPs, and G-TeaNPs examined using the MTT metabolic activity assay on HeLa cells. In all examined concentrations, G-TeaNPs presented significantly higher toxicity compared to citrate-capped AgNPs. Both types of nanoparticles were toxic to HeLa (survival <70%) starting from 0.625 mg/mL concentration. Such concentration was over 6 times higher than the working concentration of G-TeaNPs in the antibacterial assay, and over 600 times higher compared to the amount of G-TeaNPs in cocktails with phages.

**Figure S4.** Characterization of synthesized silver nanoparticles. **(a)** Size distribution profiles of green tea extract-capped AgNPs (G-TeaNPs) and control AgNPs measured by dynamic light scattering (DLS). **(b)** Zeta potential distributions for G-TeaNPs and control AgNPs.
